# Self/Co-Assembling Peptide, EAR8-II, as a Potential Carrier for a Hydrophobic Anticancer Drug Pirarubicin (THP)—Characterization and *in-Vitro* Delivery

**DOI:** 10.3390/ijms141223315

**Published:** 2013-11-26

**Authors:** Parisa Sadatmousavi, P. Chen

**Affiliations:** Department of Chemical Engineering and Waterloo Institute of Nanotechnology, University of Waterloo, Waterloo, ON N2L 3G1, Canada; E-Mail: psadatmo@uwaterloo.ca

**Keywords:** ionic-complementary, self/co-assembly, encapsulation, stability, anticancer activity

## Abstract

A short ionic-complementary peptide, EAR8-II, was employed to encapsulate the hydrophobic anticancer drug pirarubicin (THP). EAR8-II was designed to inherit advantages from two previously introduced peptides, AAP8 and EAK16-II, in their self/co-assembly. This peptide is short, simple, and inexpensive to synthesize, while possessing a low critical assembly concentration (CAC). The choice of alanine (A) residues in the peptide sequence provides moderate hydrophobic interactions, causing a minimal degree of aggregation, compared with other more hydrophobic residues. EAR8-II is an ionic-complementary peptide, similar to EAK16-II, can self/co-assemble with hydrophobic compounds such as THP, and forms a stable fibular nanostructure in aqueous solution. Physiochemical properties and cellular activities of the EAR8-II and THP complexes were evaluated and show dependency on the peptide-to-drug ratio. The complex at the peptide-to-drug mass ratio of 5:1 provides a stable solution, uniform nanostructure, and highly effective anticancer activity against various cancer cell lines. This work forms the basis for detailed studies on EAR8-II and THP formulations *in vitro* and *in vivo*, for future development of peptide-based delivery systems for hydrophobic anticancer drugs.

## 1. Introduction

**Figure 1.**
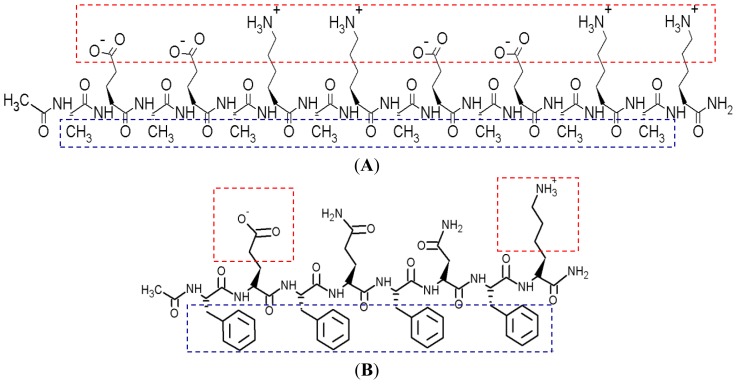

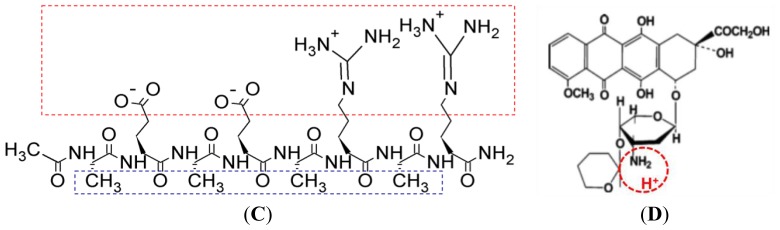
Molecular structure of (**A**) EAK16-II (Ac-AEAEAKAKAEAEAKAK-NH_2_) [4]; (**B**) AAP8 (Ac-FEFQFNFK-NH_2_) [1]; (**C**) EAR8-II (Ac-AEAEARAR-NH_2_). The red and blue box regions indicate the charged and hydrophobic residues, respectively. E: Glutamic acid (Glu); A: Alanine (Ala); K: Lysine (Lys); R: Arginine (Arg), F: Phenylalanine (Phe); Q: Glutamine (Glu); N: Asparagine (Asn); and (**D**): Pirarubicin (THP) [7].

In our recent publications, we discussed a strategy for designing amino-acid-pairing peptides, and their potential for drug and gene delivery [1,2]. A short amino-acid-pairing peptide (AAP8), with a sequence of Ac-FEFQFNFK-NH_2_, was introduced and showed promise for delivering the anticancer drug Ellipticine. Despite its practicability in anticancer drug encapsulation, AAP8 shows uncontrollable aggregation, which is not favorable for many biological applications. Therefore, a DEG-functionalization procedure was explored as a strategy to improve the multi-functionality of the nanocarrier by avoiding aggregation and to enhance bioavailability of the drug delivery system [3]. The current work focuses on a new amino-acid-pairing peptide called EAR8-II for drug delivery. This peptide, like other ionic-complementary peptides, can spontaneously organize itself into a nano/micro structure that stabilizes hydrophobic therapeutic molecules and further facilitates passive targeting [4,5].Additional advantages of using such a peptide as a carrier for drug delivery are the ease with which it can be synthesized and functionalized to incorporate long circulation, active targeting, and low immunogenic properties [6]. In particular, EAR8-II has the benefit of two well-known amino-acid-pairing peptides, EAK16-II [4] and AAP8 [1]. The design strategy exploits the ionic-complementarity of EAK16-II and the short sequence of AAP8. The shorter sequence of the peptide is the easier and less expensive synthesis procedure, and alternative charged residues in the sequence provide spontaneous self/co-assembly of the peptide with hydrophobic compounds such as Ellipticine and Pirarubicin (Figure 1).

EAR8-II (Ac-AEAEARAR-NH_2_) is a unique amphiphilic structure (Figure 1) of eight amino acids, including Glutamic acid (E), Alanine (A), and Arginine (R). Negatively charged Glutamic acid and positively charged Arginine residues are positioned in the first and second block of the sequence, providing ionic-complementarity. Alanine residues with hydrophobic side chains are located alternatively through the sequence, offering hydrophobic properties to the peptide as a whole. Self/co-assembly characteristics of this peptide arise from its ionic pairs (E and R), and hydrophobic pairs (A) that both promote self-assembly in peptides as well as co-assembly with hydrophobic molecules such as Pirarubicin (THP).

Pirarubicin (4-*O*-tetrahydropyranyladriamycin, or THP), a derivative of doxorubicin, is an anthracycline antibiotic, and acts against colorectal cancer [8], liver metastases [9], breast cancer [10], acute leukemia, and malignant lymphomas [11]. It has been reported that THP has fewer cardiotoxic effects than doxorubicin [12]. THP is applied as an anticancer drug model in the current study for the following purposes: intrinsic fluorescence property, hydrophobicity (Figure 1), promising anticancer activity, and deleterious side effects in clinical trials [12]. The fluorescence property of THP enables us to monitor the interaction of this drug with peptides and detect it in different physiological environments [7,13,14]. The low solubility of THP in aqueous environments and its possible side effects in humans highlight the important role of a delivery system for THP. Our self/co-assembling peptide-base carriers have shown characteristics to overcome problems associated with THP delivery.

The main objective of the current study is to find the effective peptide-to-drug ratio for high encapsulation efficiency, drug stabilization, and effective anticancer activity. The techniques applied are fluorescence spectroscopy, dynamic light scattering, and cellular toxicity. Emission fluorescence intensity is correlated proportionally to the concentration of encapsulated THP. Dynamic light scattering is used to determine the stability of THP in peptide solutions by measuring the average particle sizes and surface charge of peptide-drug complexes at different ratios. Anticancer activity and cellular uptake of the complexes against two cancer cells (e.g., A549 and HeLa) are also evaluated to determine the effect of peptide-to-drug ratio on therapeutic efficiency of the complex. This work provides comprehensive knowledge of the formation of peptide and drug complexes and their cellular toxicity at different ratios, paving the way for future cellular, immunological and animal studies in the development of a self/co-assembly peptide-based delivery system for hydrophobic anticancer drugs. Self/co-assembly peptide-based carriers have many advantages over other delivery systems including biocompatibility, low immunogenicity, and capability of sequence alteration based on the required interactions with therapeutic agents.

## 2. Results and Discussion

Here, we report details of the complexation between EAR8-II and Pirarubicin (THP), peptide concentration effect on the complex formation, cellular uptake, and anticancer activity of the formulation.

### 2.1. Effect of Peptide to Drug Ratio on Complex Formation

**Figure 2.**
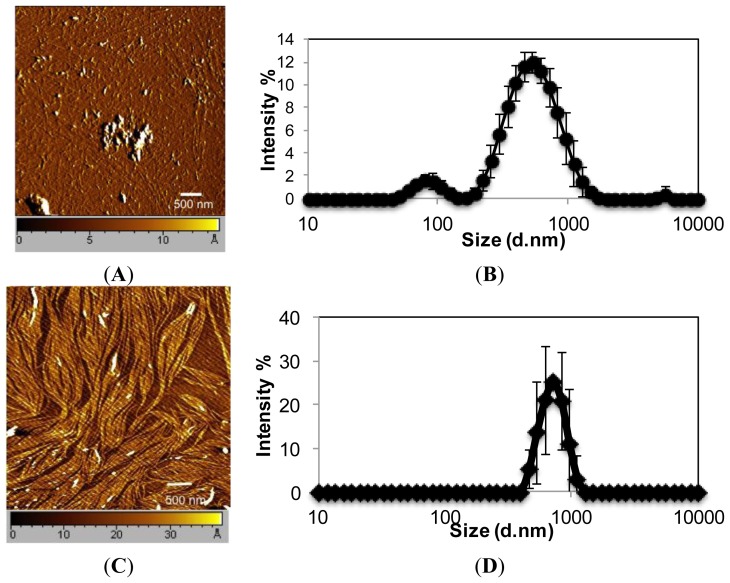
Nanostructure of (**A**) EAR8-II and (**C**) EAR8-II-THP complex by tapping mode atomic force microscopy; Intensity-based particle size distribution of (**B**) EAR8-II and (**D**) EAR8-II-THP complex in aqueous solution by dynamic light scattering. EAR8-II concentration is 0.5 mg/mL, and THP concentration is 0.1 mg/mL.

EAR8-II, similar to other ionic-complementary peptides such as EAK16-II, was observed to self-assemble into a dense fibular nanostructure with an average width and height of 10 and 15 nm, respectively. However, there was evidence of large aggregates among the fibers, which occurred due to the high concentration of peptide intermolecular interactions of amino acids. The hydrodynamic diameters of these aggregates were measured at ~500 nm, but fibers were in the range of one or two peptide monomers wide. Interestingly, EAR8-II combined with the hydrophobic drug THP forms a fibular bundle nanostructure with a hydrodynamic diameter of 712.4 ± 17.43 nm, indicating molecular interactions between the peptide and the drug called “co-assembly”. The uniformly shaped fibular structure of the peptide-drug complex provides stability and prevents uncontrollable aggregation in an aqueous solution (Figure 2).

**Figure 3.**
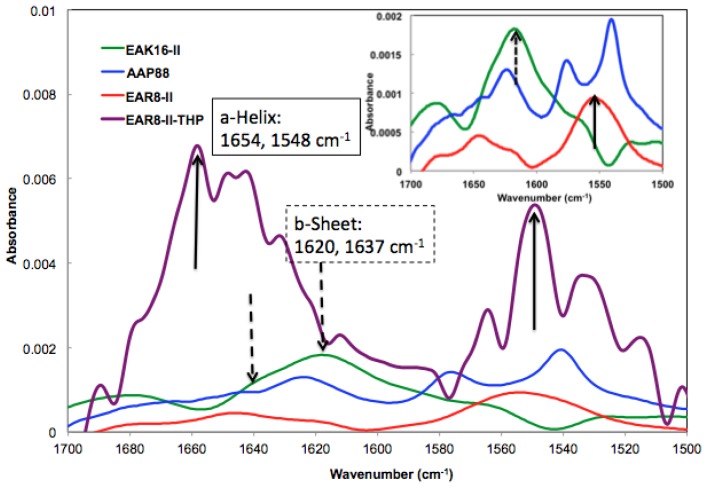
Absorbance spectrum of ( 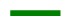) EAK16-II; ( 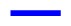) AAP8; ( 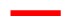) EAR8-II; and ( 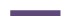) EAR8-II-THP complex collected by FT-IR indicating secondary structures.

The absorbance peaks from the FT-IR spectrum at 1614–1622 and 1630–1637 cm^−1^ correspond to β-sheets, and peaks at 1650–1658 and 1548 cm^−1^ correspond to α-helical secondary structures. As shown in Figure 3, EAK16-II formed β-sheets dominantly, and AAP8 formed a mixture of β-sheets and α-helices. However, EAR8-II showed stronger formation of α-helical structures than the other two self-assembling peptides, probably due to the length of peptide, because intramolecular interactions causing a helical structure in short sequences are stronger than the ones in longer sequences. The FT-IR spectrum collected from the complex containing EAR8-II and THP illustrated a significant increase in absorption intensity at wavenumbers corresponding to α-helical secondary (1654 and 1548 cm^−1^) structures, compared to the spectrum collected from the peptide solution. The higher absorption intensity in the FT-IR spectrum implies higher amounts of corresponding secondary structures. The helical fibular bundle structures formed in EAR8-II-THP complexes demonstrate the strong interactions between the peptide and drug to form an organized nanostructure.

**Figure 4.**
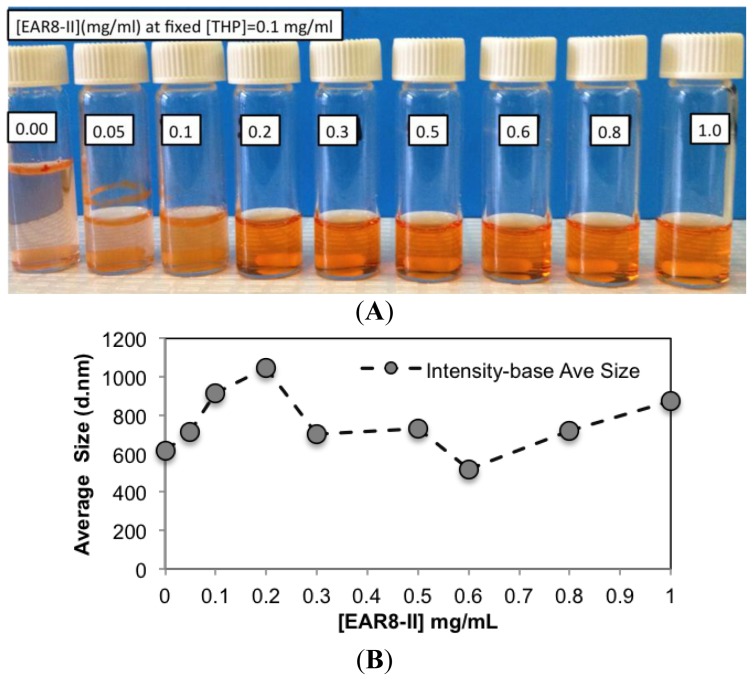
(**A**) Appearance of complexes formed by EAR8-II and THP and different EAR8-II:THP mass ratios. (From left to right: THP in water, 1:2, 1:1, 2:1, 3:1, 5:1, 6:1, 8:1, 10:1 mass ratios); (**B**) Intensity-base average hydrodynamic diameter of EAR8-II-THP complexes at fixed [THP] = 0.1 mg/mL.

To determine a suitable concentration of required EAR8-II for THP encapsulation, different concentrations of EAR8-II, in a range of 0.05–1.0 mg/mL, were prepared and combined with THP at 0.1 mg/mL concentration in aqueous solution. Solubility of THP in water is very low, whereas it can be dissolved and stabilized in an EAR8-II solution at a minimum concentration of 0.2 mg/mL. As illustrated in Figure 4A, at lower EAR8-II concentrations (0.05–0.1 mg/mL), THP molecules were not fully dissolved, and crystalline THP particles floated in the solution. However, at higher EAR8-II concentrations (0.2–0.6 mg/mL), THP molecules were fully dissolved, and consequently, a clear-transparent solution of the peptide-drug complexes formed. At much higher peptide concentrations (above 0.6 mg/mL), THP molecules were still fully soluble, but the excess amount of EAR8-II produced a cloudier solution, with clearly a higher particle size distribution. Note that amphiphilic features of EAR8-II, including the ionic and hydrophobic residues, stimulated binding with hydrophobic molecules of THP through protonation of NH_2_ to NH_3_^+^ in THP molecules and hydrophobic interactions between alanine residues and aromatic rings in the THP structure. Figure 1 shows the hydrophobic and hydrophilic regions of EAR8-II peptide, as well as protonation of the amine group in THP. Both ionic and hydrophobic interactions are the possible main interactions of EAR8-II and THP, *i.e.*, the protonation of THP in an acidic EAR8-II solution and hydrophobic interaction between THP and hydrophobic side chains of EAR8-II are the dominant forces in complexation of EAR8-II-THP.

To evaluate the characteristics of the complex formation, the change in size, pH, surface charge, and the THP fluorescence of the peptide-drug complex was monitored at different peptide concentrations. Increasing the concentration of EAR8-II made the THP become more soluble and stable in the solution. The average particle size in these complexes measured by dynamic light scattering (DLS) presented a size increase of up to [EAR8-II] = 0.3 mg/mL, followed by stabilization of the particles in the smaller range of hydrodynamic diameter ~500–700 nm, where at higher [EAR8-II] = 0.8–1 mg/mL, the solution become unstable with larger particles (Figure 4). At low concentrations of EAR8-II, non-soluble THP molecules were visible by the naked eye, and the solutions were cloudy. At concentrations of EAR8-II above 0.2 mg/mL, a uniformly suspended complex formed, where THP was fully dissolved. At a very high concentration of EAR8-II (1.0 mg/mL), peptide fibers appeared to aggregate, and the diameter measured by DLS showed a significant size increase.

**Figure 5.**
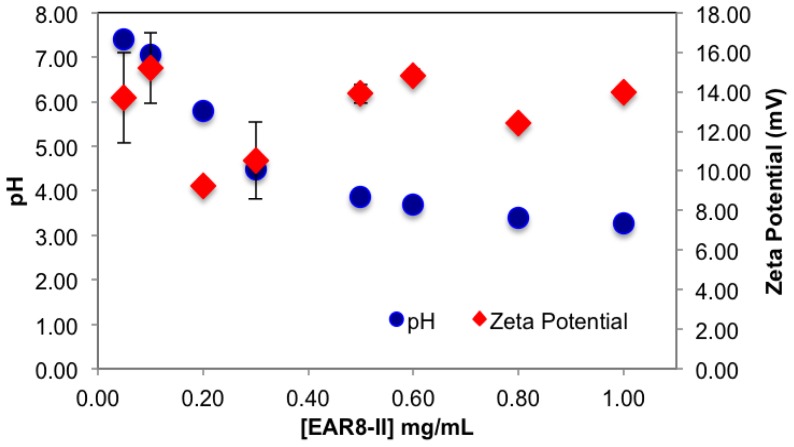
Zeta potential and pH of complex at fixed [THP] = 0.1 mg/mL and varied [EAR8-II].

The surface charge and the pH of the complex were also affected by concentration of EAR8-II. EAR8-II has an isoelectric point of ~6.55, in which at lower pH, the net charge of the peptide in aqueous solution is positive. Results showed that when the peptide concentration was increased, the pH of the solution decreased from pH 5.5 to 3.5 at a concentration of 0.05–1 mg/mL, owing to the higher [H^+^] at higher peptide concentrations. Since THP has a pKa of ~7.3 (pyridine-like nitrogen) it can be protonated in a weakly acidic environment. Consequently, acidic EAR8-II produced protonation of THP at higher peptide-to-drug ratios and led to more positive zeta potential values (Figure 5). Basically, an acidic environment provided higher [H^+^], and accordingly a higher positive surface charge reflected in zeta potential values. In Figure 5, the zeta potential values increased with increasing peptide concentration in the complex. Zeta potential values reached a plateau at EAR8-II concentrations above 0.5 mg/mL, indicating saturation of the positive charge on the surface at high peptide concentrations. Note, negatively charged glutamic acid residues in the peptide sequence may also help to stabilize the protonated THP. The high zeta potential values at very low peptide concentrations did not follow the above trend, due to the presence of non-stabilized protonated THP on the surface.

**Figure 6.**
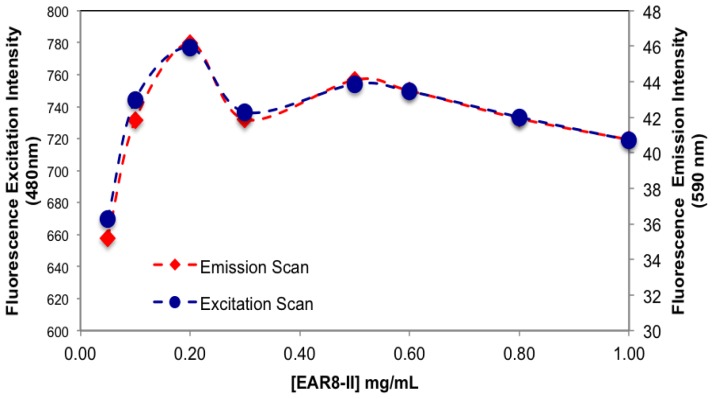
Normalized fluorescence emission and excitation intensities from EAR8-II-THP complexes at fixed [THP] = 0.1 mg/mL and varied [EAR8-II].

The amount of protonated THP in EAR8-II is also determined by measuring the fluorescence intensity of the excitation and emission by THP at different EAR8-II concentrations. Protonated THP absorbs light at 480 nm, and emits light at 590–600 nm. Fluorescence intensity for both the excitation and emission spectra is proportional to the concentration of protonated THP stabilized in the peptide solution. Although the initial concentration of THP was constant in each complex, the amount of protonated THP varied due to the dependency of the THP encapsulation efficiency on peptide concentration. As shown in Figure 6, when peptide concentration increased, the THP fluorescence intensities increased, and plateaued at peptide concentrations above 0.5 mg/mL. At lower peptide concentrations, substantial amounts of non-stabilized THP were present in the solutions, and the fluorescence intensities were significantly low, whereas at higher peptide concentrations where all the THP molecules were stabilized in the peptide aqueous environment, higher fluorescence intensities were collected. Our results showed that the minimum concentration of peptides required to fully encapsulate THP was ~0.5 mg/mL.

### 2.2. Cytotoxicity and Cellular Uptake of the Peptide-Drug Complexes

So far, we have illustrated that EAR8-II can stabilize THP in protonated form in aqueous solutions. It is expected that, due to stability and the peptide-to-drug mass ratio, different anticancer activity against cancer cells will be obtained [4,15].

Here, a series of peptide-to-drug mass-ratio complexes at a constant THP concentration of 0.1 mg/mL and EAR8-II at a range of concentrations of 0.05–1 mg/mL were employed to investigate cellular uptake and toxicity against two cancer cell lines (HeLa and A549). Cellular viability profiles for both HeLa and A549 cells at different ratios followed a similar trend, where the cell viability of A549 was relatively higher than that of HeLa, indicating more sensitivity of HeLa cells toward EAR8-II-THP complexes. In the absence of peptide in the complex, THP has been shown to induce non-significant cellular toxicity against both cell lines. Whereas, when EAR8-II stabilized THP in aqueous solution, cellular toxicity of both cells increased significantly. Due to the instability of the THP molecules in aqueous solution they do not penetrate the cell membrane effectively.

**Figure 7.**
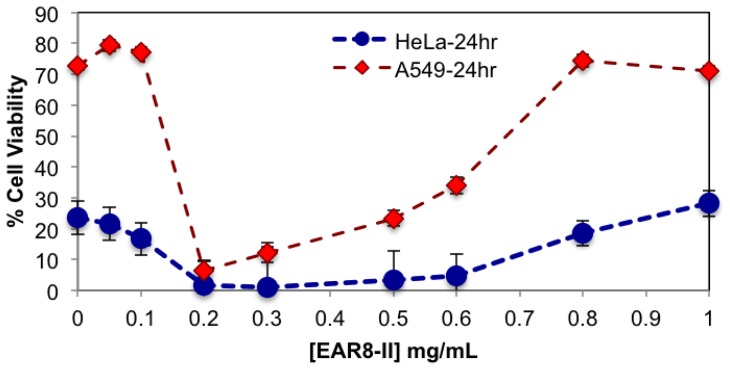
Cellular viability of HeLa and A549 cells treated with the complexes for 24 h at fixed [THP] = 0.1 mg/mL and [EAR8-II] = 0.05–1.0 mg/mL.

As shown in Figure 7, the complexes with a peptide concentration below 0.2 mg/mL and above 0.6 mg/mL had lower anticancer activity than the complexes with a peptide concentration between 0.2 and 0.6 mg/mL. Cellular viability of the complexes with an EAR8-II concentration beyond 0.2–0.6 mg/mL was significantly high, in the range of cellular viability observed in the THP control sample. This finding indicated effectiveness of EAR8-II in stabilizing THP in certain concentration ranges. As discussed above, the particle size in complexes in the 0.2–0.6 mg/mL ranges were smaller than those in other ratio complexes, which can be correlated to anticancer activity of the complexes. At lower EAR8-II concentrations, not all the THP molecules co-assembled with the peptide molecules, shown in the particle size distribution and in less efficiency toward cancer cells. However, at higher peptide concentrations, the excess presence of peptide suppressed the proficiency of THP by inhibiting the drug release effectively.

**Figure 8.**
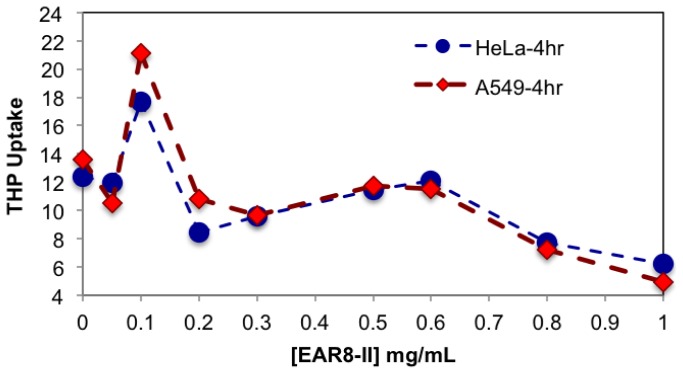
Normalized fluorescence emission intensity of THP uptake by HeLa and A549 cell lines treated with the complexes for 4 h at fixed [THP] = 0.1 mg/mL and [EAR8-II] = 0.05–1.0 mg/mL.

It is speculated that protonated THP molecules were inclined to interact with negatively charged cell membrane and promoted cellular uptake. The hydrophobic properties of THP further stimulated molecules cross the cell membrane and localized in the cytoplasm. As shown in Figure 8, cellular uptake of complexes with a peptide concentration between 0.3 and 0.6 mg/mL presented higher cellular uptake for both cell lines than the complexes beyond that ratio. The exception was the peptide-to-drug ratio 1 to 1 and the THP control, showing dramatically high uptake due to accumulation of free THP molecules on the cell membrane without them being fully localized in the cytoplasm. On the other hand, the complexes with higher EAR8-II concentrations (0.8–1.0 mg/mL) showed significantly lower cellular uptake due to highly bound EAR8-II to THP and inhibiting release of hydrophobic THP to the cell membranes.

**Figure 9.**
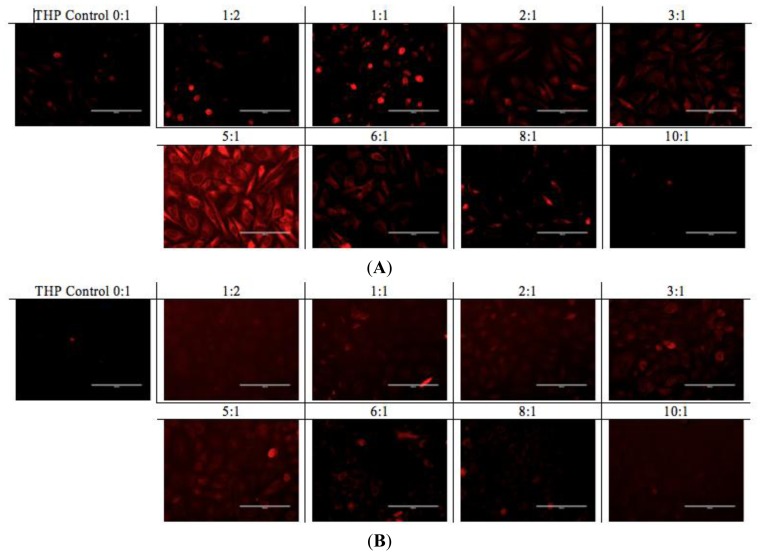
(**A**) HeLa cells; (**B**) A549 cells, treated with EAR8-II-THP at fixed [THP] = 0.1 mg/mL and varied [EAR8-II]. The ratios between EAR8-II to THP are indicated above each image. EAR8-II concentrations: (0:1) = 0.0 mg/mL; (1:2) = 0.05 mg/mL; (1:1) = 0.1 mg/mL; (2:1) = 0.2 mg/mL; (3:1) = 0.3 mg/mL; (5:1) = 0.5 mg/mL; (6:1) = 0.6 mg/mL; (8:1) = 0.8 mg/mL; (10:1) = 1.0 mg/mL. Scale bar is 100 μm.

A similar pattern was observed in the fluorescence microscopy images from the cells treated with EAR8-II-THP complexes at different ratios. For both cell lines, complexes with peptide concentrations of 0.3 and 0.5 mg/mL resulted in the highest cellular uptake of THP in cytoplasm. In contrast, complexes at lower and higher concentrations of the peptide showed lower localization of THP in cytoplasm. Note that the bright red fluorescence observed in 1:2 and 1:1 ratio complexes were from non-dissolved THP not localized in cytoplasm. At the ratios of 3:1 and 5:1, the localization of THP in cytoplasm was very uniform, whereas other ratio complexes had THP randomly localized or just accumulated on the membrane (Figure 9). These results led us to select appropriate formulations to treat different cancer cells. So far, from the cumulative characterization and cellular uptake and toxicity results, the complex with the peptide-to-drug ratio of 5:1 has been shown to be a more stable and efficient solution for drug delivery, because of its high encapsulation efficiency, uniform nanostructure, relatively high cellular toxicity and uptake.

**Figure 10.**
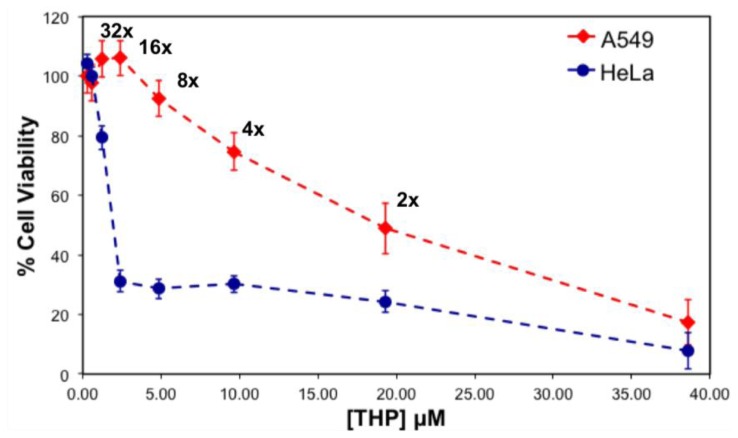
Viability of A549, HeLa cells treated with serially diluted complexes at 5:1 EAR8-II to THP complex for 24 h.

Au *et al.* emphasized the importance of drug stability upon dilution during treatment time in clinical trials [16]. Since, the peptide-drug complex at a 5:1 ratio illustrated appropriate properties such as size, charge and toxicity against cancer cells, cytotoxicity testing was performed for this particular complex, and its serial dilution solutions were tested against two human cancer cell lines, including the non-small lung cancer cell A549, cervical adenocarcinoma HeLa. Figure 10 represents the viability of the cells treated with the 5:1 ratio complex and its serial dilution in water, where the final concentration of THP is between 0.3 and 38 μM. The IC_50_ values of the complex for the cancer cells were calculated, and followed the following trend; THP concentration at ~19.3 μM for A549 >~1.8 μM for HeLa cells [17].

Toxicity results for both serially diluted and variable peptide-to-drug ratio complexes revealed relatively higher cell viability of A549 compared to HeLa cells, denoting that A549 cells are highly resistant towards the EAR8-II-THP complex. Similar results were observed previously for EAK16-II and EPT complexes against MCF-7 cells and A549, in which A549 cells showed higher resistance than other tested cells [4]. The reason for different levels of toxicity of the same complex towards various cancer cells is not fully clear, but it might arise from different reactions of cancer cells to the EAR8-II-THP complex.

Note that the cell viability of the THP control solution (no peptide) for both types of cell lines was significantly higher than for the respective THP concentrations in the presence of EAR8-II. For THP at 38 μM in water, the cellular viability was 64.9 ± 6.4, 29.58 ± 3.69 (%) for A549, HeLa cells, respectively. These findings clarify the important role of EAR8-II in stabilizing THP in an aqueous environment, and consequently delivering it into cells and causing cellular toxicity.

In the case of a stable complex upon dilution, the increase in cellular viability happens smoothly and gradually due to the lower drug concentration. Based on the observed cell viability results at 24 h incubation time, the viability of A549 increased gradually, when the complex was diluted. In contrast, the viability trend for HeLa cells did not increase as smoothly as for A549 cells. The increase of HeLa cell viability after sixteen times dilution was sharp, indicating instability of the complex at high dilution factors in water, in the presence of a medium. One reason for complex instability is the instability of protonated THP in a highly diluted complex. As discussed, protonated THP is stabilized in peptide environments at low pH. Higher amounts of water lead to increased pH and consequently lower THP stability in solution. This effect might also arise from the stability of the complex in the different culture media used for different cell lines. The content of each culture medium varied, and THP might have distinct properties in each medium. Further experiments are required to confirm this phenomenon. Overall, EAR8-II-THP complexes at a peptide-to-drug ratio of 5:1 demonstrated promising results against cancer cells and stabilized in the cell culture environment.

## 3. Experimental Section

### 3.1. Materials

The EAR8-II (Ac-AEAEARAR-NH_2_) peptide with a molecular weight of 913.94 g/mol and a purity of >75% was synthesized in our laboratory by the solid-phase peptide synthesis (SPPS) method [18]. The *N*-terminus and *C*-terminus of the peptides were protected by acetyl (COOCH_3_) and amine (NH_2_) groups, respectively. The molar mass of this peptide was verified by Matrix-assisted laser desorption ionization time of flight mass spectroscopy (Q-TOF Ultima Global, Waters, Milford, MA, USA). The anticancer drug Pirarubicin (THP) (AB2000083), with the commercial name of THP and molecular weight of 627.63 g/mole, was purchased from ABBLIS (Houston, TX, USA).

All cell lines, purchased from Cedarlane (Burlington, ON, Canada), were used in the current study and cultured in their respective culture medium as follows: Human Cervical Adenocarcinoma: HeLa (CCL-2) in Eagle’s Minimum Essential Medium (EMEM) (ATCC30-2003), Human lung cancer: A549 (CCL-185) in F-12 medium (ATCC30-2004). All medium solutions were prepared with 10% fetal bovine serum (FBS-F1051).

### 3.2. Methods

#### 3.2.1. Sample Preparation

EAR8-II peptide solutions in the concentration range of 0.05–2 mg/mL (0.0547–2.188 mM) were prepared by dissolving peptide lyophilized powder in Milli-Q (18.2 MΩ) water (EMD Millipore, Billerica, MA, USA) followed by bath sonicating for 10 min. Freshly prepared peptide solutions were added to THP to reach fixed [THP] concentrations at 0.1 mg/mL (0.159 mM), followed by mechanical stirring at 900 rpm for 24 h.

#### 3.2.2. Dynamic Light Scattering (DLS)

The particle size distribution and zeta potential of peptide-drug complexes at different peptide-to-drug ratios were measured by a Zetasizer Nano ZS (Malvern Instruments, Worcestershire, UK). Appropriate settings, viscosity, refractive index, and dispersant solvent were set for each measurement at 25 °C. A small 50 μL volume of the sample was transferred from the vial to a disposable solvent resistant micro cuvette (ZEN0040, Malvern, Worcestershire, UK). The scattered light intensities of the samples were collected at an angle of 173°. This was known as backscatter detection, *i.e.*, the relationship between the size of a particle and its scattered light intensity obtained with the multimodal Contin algorithm, which was provided in the Dispersion Technology Software 5.1 package (Malvern Instruments, Worcestershire, UK).

For zeta potential measurements, the sample was injected into a 1 cm × 1 cm disposable cuvette with a volume of ~1 mL, and a dip cell (ZEN1002) was inserted into the solution so that the solution covered the electrodes on the dip cell. The zeta potential distribution was directly calculated from electrophoretic mobility distribution based on the Smoluchowski formula. For each solution, the zeta potential was repeated three times. The values reported herein correspond to an average of the peak values of the zeta potential distribution from replicates.

#### 3.3.3. Steady-State Fluorescence Emission

The THP fluorescence emission spectra were acquired on a photon technology international spectrafluorometer (Type QM4-SE, London, ON, Canada) with a continuous xenon lamp as the light source. The emission spectra were collected in the wavelength range of 500–800 nm, while samples were excited at 480 nm. The maximum emission peak of THP is at a wavelength of 590–600 nm. The excitation spectrum was collected in the wavelength range of 200–500 nm. The maximum excitation peak of THP is at wavelength of 470–490 nm. The excitation and emission slit widths of the monochromators, which control the amount of light coming in and out of the sample chamber, were set at ½ and 2 turns to yield a spectral resolution of 1 and 4 nm, respectively. The fluorescence intensity at 590 and 480 nm for emission and excitation were obtained by taking the average of twenty points of fluorescence intensity, respectively. Each fluorescence spectrum was divided by lamp intensity to account for eventual lamp fluctuations [19]. The normalized fluorescence intensity for both emission and excitation spectra at 590 and 480 nm was plotted for various peptide-to-drug ratios.

#### 3.3.4. Cellular Toxicity and Uptake

Two human cancer cell lines, with 10^4^ cells per well concentrations, were seeded in 96-well plates, followed by treatment with different peptide-drug complex ratios for 24 h. Cellular-toxicity experiments were performed with sensitive colorimetric assay called Cell counting kit-8 (CCK-8) (Dojindo Molecular Technologies, Inc. Burlington, ON, Canada). CCK-8 contains highly water-soluble tetrazolium salt (WST-8), which is reduced by dehydrogenase activities in cells to give the yellow color of the formazan dye, which is soluble in culture media. The amount of formazan dye is measured by absorption (O.D.) at 450 nm, which is directly proportional to the number of living cells.

Cellular uptake of THP was also carried out by spectroscopy, exploiting the intrinsic fluorescence properties of THP (excitation wavelength: 480 nm, emission wavelength: 590 nm). Cells were plated in 12-well plates, with concentrations of 2 × 10^5^ cells per well. They were then treated with peptide-drug complexes for 2–4 h. For spectroscopy with the PTI system, after 6 h incubation at 37 °C, the cells were lysed with 1% SDS in Tris-EDTA, followed by probe sonicating to complete cell lysis. The lysed cell suspensions were excited at 480 nm, and emission spectra were collected from 500 to 800 nm to quantify the THP content of the treated cells. Each spectrum was corrected by spectra of lysed untreated cells. The amount of drug uptake is directly proportional to the emission peak intensities. Higher fluorescence emission intensity indicates higher cellular uptake of THP.

## 4. Conclusions

The current article introduced a new self/co-assembling ionic-complementary peptide, EAR8-II, stabilizing the hydrophobic drug pirarubicin (THP) in protonated form in aqueous environments, as shown by surface charge and fluorescence measurements. Results show the complex formation to be peptide-to-drug ratio dependent. Increasing EAR8-II concentration at a constant THP concentration reduced the pH value of the complex and consequently caused protonation of THP stabilized in solution. At lower peptide-to-drug ratios, an insubstantial amount of THP molecules were solubilized in aqueous solution, according lower fluorescence emission intensity than in complexes with higher peptide-to-drug ratios, in which more THP is stabilized. The peptide-to-drug ratio affected the particle size distribution within a complex; where at very low and very high concentrations of EAR8-II, the average particle size was large, indicating the presence of long fibers and large aggregates in the solution. However, in complexes with peptide concentrations between 0.2 and 0.6 mg/mL, the average particle size observed was ~600 nm. Cytotoxicity and cellular uptake by cancer cell lines demonstrated peptide-to-drug ratio dependent behavior, in which, at very low and very high ratios, the anticancer activity was lower than that of peptide-to-drug mass ratios between 2:1 and 5:1. Deficiency and excess of the peptide in stabilizing THP caused not fully encapsulated and highly trapped THP molecules, respectively. The complex at a 5:1 peptide-to-drug mass ratio was recognized as an optimum peptide-to-drug ratio complex where the highest encapsulation efficiency was observed for the lowest peptide concentration present in the complex. This complex also offered the highest anticancer activity and cellular uptake against cancer cells compared to other ratio complexes. The anticancer activity of this complex and its serial diluted solutions were further studied against two cancer cells. The IC_50_ of THP was calculated as ~19.3 μM for A549 >~1.8 μM for HeLa cells, indicating sensitivity of HeLa cells compared with A549 toward EAR8-II-THP complexes. This study provides a platform for using self/co-assembly peptides as carriers for hydrophobic drugs in future for *in vitro* and *in vivo* applications by introducing a stable complex with relatively high therapeutic efficacy against cancer cells.
